# Real-Time Cycle Slip Detection in Single-Frequency GNSS Receivers Using Dual-Index Cross-Validation and Elevation-Dependent Thresholding

**DOI:** 10.3390/s25196162

**Published:** 2025-10-04

**Authors:** Mireia Carvajal Librado, Kwan-Dong Park

**Affiliations:** 1 Department of Electrical and Computer Engineering, Inha University, 100 Inha-ro, Incheon 22212, Republic of Korea; mireiacl@inha.edu; 2 PP-Solution Inc., 606 Seobusaet-gil #B-2311, Seoul 08504, Republic of Korea; 3 Department of Geoinformatic Engineering, Inha University, 100 Inha-ro, Incheon 22212, Republic of Korea

**Keywords:** GPS, GNSS, cycle slip, single-frequency, real-time

## Abstract

Cycle slips, abrupt discontinuities in carrier-phase measurements, pose a significant challenge for single-frequency GNSS receivers, particularly in real-time applications where rapid detection is critical. Unlike dual-frequency approaches, these receivers cannot rely on redundant combinations to isolate slips from other errors. This study proposes a real-time cycle slip detection algorithm for single-frequency GNSS receivers based solely on short-term differencing of pseudorange and carrier-phase observables. The method employs a two-step logic: first-order differencing of code-minus-carrier and second-order differencing of carrier phase. Both steps employ satellite elevation-dependent adaptive thresholds, enabling robust detection under diverse signal conditions. The method requires no user position, receiver-generated tracking flags, or additional sensor data. Experimental results reveal that the algorithm achieves over 98% detection accuracy for slips exceeding 10 cycles, with no false positives in artificial slip testing, and 87.93% agreement with Loss of Lock Indicators (LLI) during periods in which the receiver indicated signal instability.

## 1. Introduction

Continuous tracking of carrier-phase observables forms the backbone of precise global navigation satellite system (GNSS) positioning techniques, including real-time kinematic (RTK), precise point positioning (PPP), and geodetic surveying. These techniques can achieve centimeter-level positioning accuracy when integer ambiguities are correctly resolved and maintained throughout the observation period [1,2]. However, carrier-phase measurements are vulnerable to abrupt cycle slips, which are integer jump discontinuities caused when the receiver loses its phase lock due to signal obstruction, multipath, weak signals, or receiver dynamics. These slips can range from a few to hundreds of cycles, depending on the severity and duration of the signal interruption. Such disruptions corrupt the carrier-phase observable and degrade ambiguity resolution integrity [3,4,5].

Cycle slip detection represents a critical preprocessing step in high-precision GNSS applications. Undetected slips invalidate previously resolved integer ambiguities and force re-initialization of the positioning solution, causing convergence delays that can extend from minutes to hours depending on the constellation geometry and atmospheric conditions [6]. Such delays significantly degrade accuracy until satellite geometry changes sufficiently to enable rapid re-convergence. The economic and operational implications of such positioning failures are substantial in applications such as precision agriculture, autonomous vehicle navigation, surveying, and construction, where continuous high-precision positioning is essential for system reliability and safety.

Dual-frequency receivers benefit from ionosphere-free combinations and specialized observables such as the Melbourne-Wübbena combination for robust cycle slip detection and repair [7]. In contrast, single-frequency systems face significant challenges due to the lack of redundant frequency measurements that can effectively isolate cycle slips from other error sources [8]. The TurboEdit algorithm, which combines Melbourne-Wübbena and geometry-free observables, has been extensively applied in dual-frequency systems for its ability to detect and repair cycle slips, mitigate insensitive cycle slips, and deliver robust detection accuracy [9]. TurboEdit leverages dual-frequency measurements to automatically identify and correct cycle slips in real-time by operating on undifferenced carrier-phase data while remaining robust to clock and tropospheric variations [10]. Recent works have also refined TurboEdit by introducing ionosphere-free combinations and polynomial fitting, enhancing sensitivity to small slips under challenging conditions [11]. This provides useful context for contrasting our single-frequency framework with dual-frequency approaches.

However, single-frequency receivers lack such redundant measurements; therefore, they must rely on alternative cycle slip detection approaches, such as polynomial fitting to model phase evolution, geometry-based residual analysis that compares observed and predicted phases, and time-differenced observables that highlight sudden phase discontinuities [12,13,14,15]. However, the performance of these methods is often constrained by noise that varies significantly with satellite elevation angles, environmental conditions, and receiver quality [16]. With only one frequency, distinguishing true cycle slips from measurement noise, multipath, and atmospheric disturbances becomes challenging with poor signal reception, particularly in urban environments where multipath and signal blockage are prevalent.

Amongst typical single-frequency cycle slip detection approaches, the code-minus-carrier combination has proven effective due to its high sensitivity to phase discontinuities and relative insensitivity to atmospheric effects over short time intervals [17,18]. Building on this foundation, several specific methodologies have emerged for single-frequency cycle slip detection.

Another commonly used method, known as Time-Differenced Carrier Phase (TDCP), involves the time differencing of carrier-phase observations. This technique cancels integer ambiguities through epoch-to-epoch differences for velocity estimation and cycle slip detection. Research on enhanced-grade receivers and smartphones reveals that TDCP yields millimeter-per-second velocity accuracy and can detect cycle slips as brief as one epoch [19]. However, its performance degrades markedly in noisy, low-cost devices. To overcome this issue, urban multipath mitigation systems have employed TDCP with inertial navigation systems (INS) to reach sub-decimeter accuracy [20].

Alternative approaches to INS have been developed to address TDCP limitations. Doppler-Aided Cycle Slip Detection (DACSD) compares changes in carrier-phase with integrated Doppler-derived ranges. Cycle slips appear as inconsistencies between these two measurements. While Doppler is immune to slips, its high noise complicates the detection of small slips [21]. Studies using unmanned aerial vehicle (UAV) platforms have combined Doppler measurements with velocity and clock-drift estimations from dual-frequency data to reduce test statistic noise. However, these methods have limited applicability in pure single-frequency systems [22].

Geometry-based (GB) strategies represent another class of detection methods that can be used for single-frequency receivers. These leverage satellite-receiver range predictions, often using INS-aided Kalman filtering, to detect slips by analyzing residuals or covariance estimates [23]. Such geometry-based methods often deliver better detection accuracy but traditionally require multi-satellite redundancy and environmental modeling.

Statistical and model-based approaches further enhance detection robustness, employing polynomial fitting and statistical hypothesis testing on time-windowed or differenced measurements to identify anomalous behavior. Bayesian spline-fitting and least-squares polynomial regression on phase-minus-code residuals have successfully identified slips even in disturbed environments [24]. More sophisticated implementations combine multiple techniques to achieve detection resolutions of one cycle even under severe ionospheric activity [25].

Newer hybrid methods aim to preserve single-frequency simplicity while improving resilience. Bae et al. achieved sub-meter accuracy in urban vehicle navigation using Time-Differenced Code-Minus-Carrier with INS assistance and multipath screening [26]. Recent standalone GNSS chip developments have employed novel dual-board designs sharing one antenna to form between-epoch, cross-board, and cross-satellite statistics. This mechanism eliminates reference dependency and mitigates clock errors, enhancing slip detection and correction under multipath and dynamic sampling conditions [27].

Despite these advances, key limitations persist. Doppler-based methods struggle with small slips; geometry-informed or IMU-assisted schemes introduce complexity, cost, or dependency on external sensors; polynomial fitting may require manual tuning or offline processing; and common elevation-static thresholds adapt poorly across diverse environments. There is still a need for a purely single-frequency, measurement-only, real-time method that adapts dynamically to environmental factors and maintains robustness across different scenarios.

Therefore, this study proposes a two-step cycle slip detection algorithm tailored for real-time applications using only single-frequency GNSS measurements. The approach integrates the first-order time difference in the code-minus-carrier with the second-order time difference in the carrier-phase observables. The algorithm initially identifies peaks in the first-order time difference in the code-minus-carrier, which are then validated by corresponding peaks in the second-order time difference in the carrier-phase observables. This cross-verification reduces false detections from multipath, code noise, and environmental dynamics.

A key feature of the method is its integration of elevation-dependent thresholds, derived from empirical modeling of residual variability across different satellite geometries and environments. Additional refinements include detrending and robust statistical processing to handle non-stationary noise and low signal-to-noise ratio (SNR) conditions. The algorithm requires neither user position estimates nor manufacturer-specific tracking flags, making it adaptable to lightweight or embedded implementations.

The proposed algorithm was validated under both static open-sky environments and forested conditions with considerable signal obstruction. Performance was assessed using both natural and controlled cycle slips. The controlled cycle slips are artificially injected cycle slips that are used to evaluate detection sensitivity and false alarm rates. Comparative analysis with traditional Loss of Lock Indicators (LLI) provides additional context for practical implementation.

While first-order differencing of the code-minus-carrier and second-order differencing of the carrier phase are well-known tools, their combined use in a two-step cross-validation scheme, supported by elevation-dependent thresholding, has not been systematically studied for single-frequency receivers. This study, therefore, contributes a framework that integrates these elements and validates its robustness through both natural and large-scale artificial slip tests.

The remainder of this article is structured as follows. Section 2 presents the data acquisition strategy. Section 3 details the mathematical framework and algorithmic design. Section 4 describes the experimental analysis and results. Finally, Section 5 outlines the key conclusions of this study.

### Key Contributions

The contributions of this study can be summarized as follows:

- Introduction of a dual-index cross-validation framework that integrates first-order time-differenced code-minus-carrier with second-order time-differenced carrier-phase for reliable cycle slip detection in single-frequency receivers.
- Development of elevation-dependent adaptive thresholds to account for noise variations across different satellite geometries.
- Comprehensive validation using both natural and artificial slips (>1000 test cases), demonstrating robustness in open-sky and obstructed environments.

## 2. Experimental Setup and Data Acquisition

To develop and validate the proposed cycle slip detection algorithm, single-frequency GNSS datasets were collected under different conditions and environments. These datasets were used both for empirical modeling and performance evaluation.

Observations were recorded using a u-blox EVK-M8T receiver module (u-blox AG, Thalwil, Switzerland) configured through the u-center v24.10 software (u-blox AG, Thalwil, Switzerland), logging raw carrier-phase measurements (L1), pseudorange data, and navigation messages at a sampling rate of 1 Hz. To ensure consistency across all test environments, only Global Positioning System (GPS) and Galileo constellation signals were processed, denoted in Section 4 as GXX and EXX, respectively, where XX signifies their respective Pseudorandom Noise (PRN) numbers.

Data were collected under two distinct operational scenarios to represent various practical conditions encountered in GNSS applications:

- Static Open-Sky (OS): Static measurements were collected at a sports field with nearly unobstructed sky visibility, minimal multipath interference, and open terrain conditions. These datasets served as reference cases for stable reception under optimal signal conditions.
- Static Forested (FR): Static observations were recorded in a park with partial canopy coverage in wooded areas within an urban area. These datasets represent challenging conditions characterized by degraded signal quality, increased multipath propagation, and rapid signal strength fluctuations due to foliage-induced scattering.

**Figure 1:**
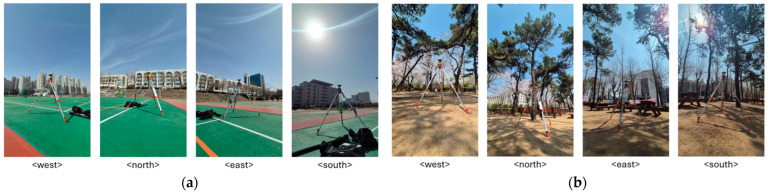
Surrounding views at the static (**a**) OS and (**b**) FR test sites.

To visually document sky visibility, panoramic photos were captured in all four directions at both static test sites (Figure 1). These images illustrate the difference in visibility and obstruction between the FR and OS scenarios. The FR site’s partial canopy and nearby structures limited sky visibility, particularly toward the north and east, whereas the OS site provided clear satellite visibility across all azimuths.

Individual recording sessions lasted one hour. Data collection sessions were distributed across different seasons and times of day to capture varying satellite geometries and environmental conditions. The OS datasets were acquired on 10 December 2024 (14:00–15:00 KST), 11 March 2025 (12:50–13:50 KST), 20 March 2025 (11:40–12:40 KST), and twice on 28 March 2025 (10:45–11:45 KST and 11:50–12:50 KST). The FR datasets were collected on 10 December 2024 (14:00–15:00 KST), 7 March 2025 (15:40–16:40 KST), 11 March 2025 (11:25–12:25 KST), and 16 April 2025 (11:25–12:25 KST and 12:30–13:30 KST).

## 3. Methodology

### 3.1. Observation Model and Detection Indices

The proposed cycle slip detection methodology is based on analyzing derived GNSS observables that amplify temporal discontinuities while suppressing gradual variations. Carrier-phase measurements ϕ (meters) and code phase measurements P (meters) are generally expressed using standard GNSS functional models [6] as follows:

$$\phi =\rho +c\left(dT-dt\right)+\;T-I+\lambda N+\epsilon_{\phi }$$

$$P=\rho +c\left(dT-dt\right)+T+I+\epsilon_{P}$$

where ρ denotes the instantaneous satellite-receiver range (m); c represents the speed of light in vacuum (m/s); dT and dt signify the receiver and satellite clock offsets for a given observable (s), respectively; T refers to the tropospheric delay (m); I corresponds to the ionospheric delay on $L_{1}$ (m); λ denotes the carrier wavelength (m); N is the integer carrier-phase ambiguity (cycles); and ϵ reprsesents unmodeled quantities such as noise and multipath.

From these two measurements, the Code-Minus-Carrier ($C^{-}$) can be computed as part of the detection methodology and is constructed by differencing code and carrier-phase measurements as follows:

$$C^{-}=P-\phi =2I-\lambda N+\epsilon_{C^{-}}$$

To enhance the detectability of cycle slips, time-differencing is applied. Time-differencing computes the difference between consecutive epoch measurements separated by a time interval Δt, isolating sudden changes from gradual variations in the observables. When cycle slips occur, they create an abrupt step change in the carrier-phase ambiguity. Time-differencing transforms these step changes into sharp peaks that are easily detectable, while gradual atmospheric or geometric variations are substantially suppressed, assuming no significant ionospheric disturbances are present. The use of time-differencing for cycle slip detection is not new and has been extensively described in GNSS literature [12,13,14,15,26], primarily due to its ability to enhance the visibility of cycle slips.

The algorithm employs two time-difference detection indices: the first-order time-differenced code-minus-carrier ($\Delta_{t}C^{-}$) and the second-order time-differenced carrier-phase ($\Delta_{t}^{2}\phi$), which provide complementary information about potential cycle slip events. First order and second order time differencing are denoted as $\Delta_{t}$ and $\Delta_{t}^{2}$, respectively. The two detection indices are computed as follows:

$$\Delta_{t}C^{-}=C^{-}\left(t+\Delta t\right)-C^{-}\left(t\right)=2\Delta_{t}I-\lambda \Delta_{t}N+\epsilon_{C^{-}}$$

$$\Delta_{t}^{2}\phi =\Delta_{t}\phi \left(t+\Delta t\right)-\Delta_{t}\phi \left(t\right)=\Delta_{t}^{2}\rho +c(\Delta_{t}^{2}dT-\Delta_{t}^{2}dt)+\Delta_{t}^{2}T-\Delta_{t}^{2}I+\lambda \Delta_{t}^{2}N_{i}^{j}+\epsilon_{\phi }$$

The $\Delta_{t}C^{-}$ index exhibits sensitivity to ionospheric delay changes, code multipath effects, and ambiguity terms. Due to the sensitivity to the ambiguity terms, cycle slips appear as step functions in N, producing corresponding impulses in the time-differenced observable. This index serves as the primary screening tool for cycle slip detection. In contrast, the $\Delta_{t}^{2}\phi$ index primarily reflects second-order variations in geometry, clock terms, atmospheric delays, and ambiguity terms. This index is less affected by code-related noise, making it highly effective as a confirmation step for the cycle slip detections.

The detection approach leverages the different signal error responses to the two indices: true cycle slips produce simultaneous peaks in both $\Delta_{t}C^{-}$ and $\Delta_{t}^{2}\phi$. Code noise or multipath typically affects only $\Delta_{t}C^{-}$, with minimal impact on $\Delta_{t}^{2}\phi$, and atmospheric or geometric variations impact both weakly and usually not simultaneously.

This differential sensitivity to error sources forms the basis for the cross-validation logic, enabling robust detection. A candidate slip is confirmed only when both indices exceed their respective thresholds at the same epoch, minimizing false detections from non-slip-related disturbances.

### 3.2. Two-Step Detection Algorithm

From the detection indices in Section 3.1, the proposed detection algorithm follows a two-step logic combining the behaviors of $\Delta_{t}C^{-}\;$ and $\Delta_{t}^{2}\phi$ to identify true cycle slips while minimizing false positives from multipath, receiver noise, or gradual signal drift. The method operates independently for each satellite signal without external sensors.

In the first step, candidate epochs are flagged as possible cycle slips if the $\Delta_{t}C^{-}$ index exceeds a satellite-specific threshold, dynamically scaled according to satellite elevation angle to account for signal quality degradation at low elevation angles (Section 3.3). Candidate epochs are then checked in the confirmation step to assess whether the corresponding $\Delta_{t}^{2}\phi$ value also exceeds its threshold. This cross-validation logic is summarized in Table 1.

**Figure 2:**
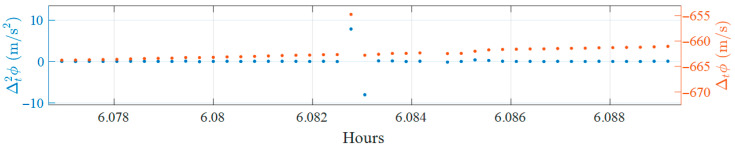
Mirrored peaks introduced by second-order time-differencing in carrier phase data.

Preliminary experiments revealed that the second-order time-difference operation can produce a spurious peak occurring one epoch after a suspected cycle slip, as shown in Figure 2. This figure corresponds to a data fragment collected in static conditions on Day of Year (DOY) 345 of 2024 in the FR environment.

In the example, a possible cycle slip appears as a peak in the first-order time difference in the carrier phase (orange) near 6.083 h. The second-order time difference (blue) shows a peak at the same epoch but also a peak of inverse sign and similar magnitude in the following epoch. This “mirrored” peak is not caused by a real slip; it is a mathematical artifact of the second-order differencing process.

To suppress these artifacts, the algorithm filters mirrored peaks from the $\Delta_{t}^{2}\phi$ series by comparing the current and previous $\Delta_{t}^{2}\phi$ values. If a peak is followed by another of similar magnitude but opposite sign, it is considered a byproduct of the differencing operation. Ideally, this ratio is −1, but due to measurement noise, a tolerance is allowed. This tolerance or range is decided empirically. Therefore, peaks are excluded when the ratio of $\Delta_{t}^{2}\phi \;\left(t\right)\;$ to $\Delta_{t}^{2}\phi \;(t-1)$ falls within the following range:

$$\frac{\Delta_{t}^{2}\phi \;(t)}{\Delta_{t}^{2}\phi \;(t-1)}\in [-1.5,\;-0.65]$$

Only peaks outside this tolerance range are retained as valid cycle slip candidates, eliminating systematic artifacts caused by second-order differencing.

**Figure 3:**
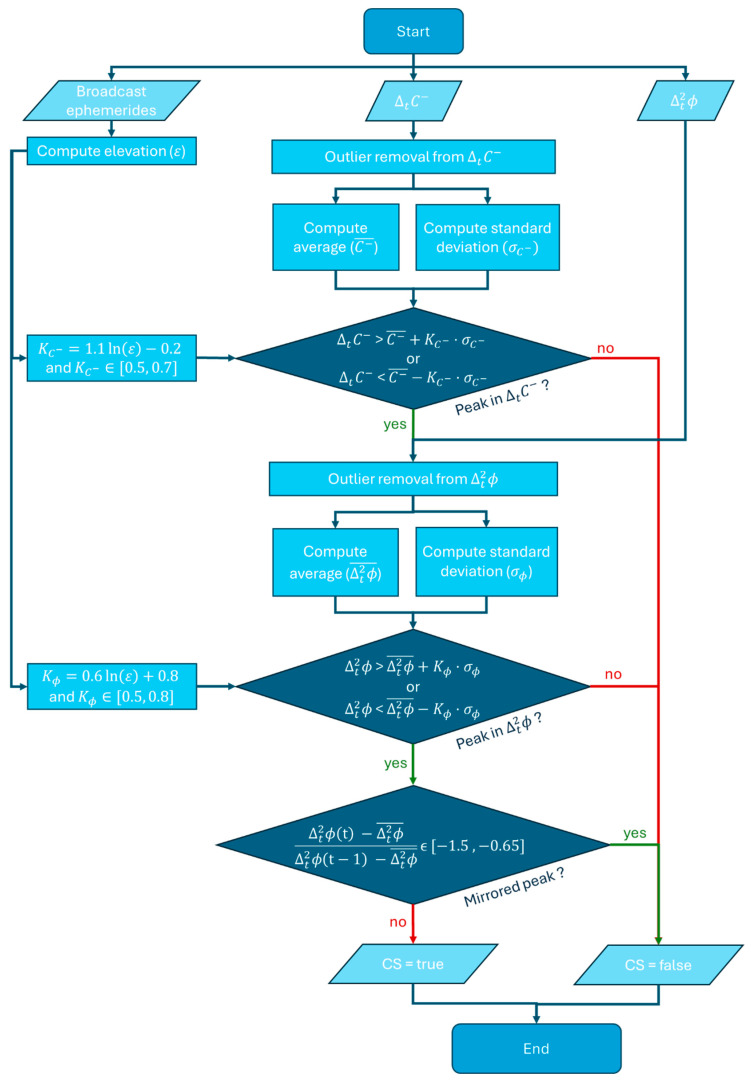
Flowchart of the proposed cycle slip detection framework, combining dual-index cross-validation with mirrored-peak suppression, elevation-dependent thresholds, and outlier handling.

The overall algorithm logic is summarized in Figure 3. The flowchart illustrates the two-step cross-validation logic together which begins with the computation of satellite elevation from broadcast ephemerides, which are used to determine the elevation-dependent thresholds. Outlier removal is first conducted on $\Delta_{t}C^{-}$, followed by the calculation of its mean and standard deviation. These statistics are used to identify peaks that may correspond to potential cycle slips. Candidate events are then validated against $\Delta_{t}^{2}\phi$, where a second usage of outlier handling and thresholding is applied. To mitigate false detections caused by symmetric fluctuations, a mirrored peak suppression step is included. A cycle slip is declared only when both indices consistently indicate an anomaly under the defined thresholds. While some of these elements are explained in subsequent subsections, they are included here to provide a complete overview of the algorithm pipeline.

### 3.3. Elevation-Dependent Thresholding

Both detection indices rely on thresholds to identify peaks. Because measurement noise varies significantly with satellite elevation, especially in low-cost GNSS receivers, the thresholds are dynamically adjusted to reflect elevation-dependent signal quality. Accordingly, the values of $\Delta_{t}C^{-}$ and $\Delta_{t}^{2}\phi$ are related to the elevation angle, with their thresholds established by empirical curves derived from residuals across multiple datasets.

**Figure 4:**
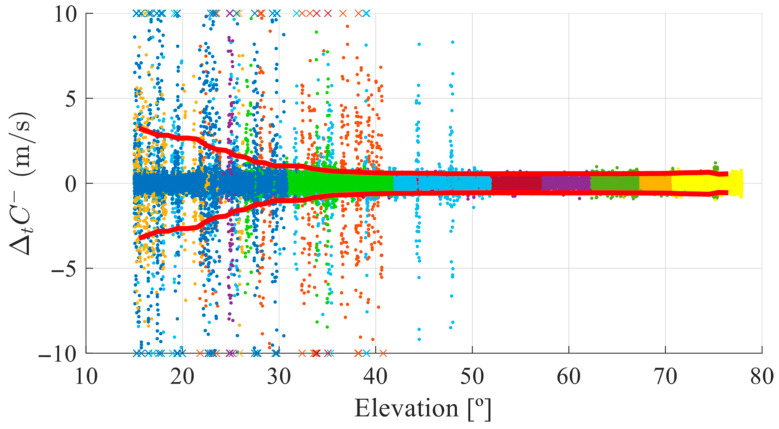
Scatter plot of $\Delta_{t}C^{-}$ vs. elevation in FR on DOY 345, illustrating increased dispersion at low elevations and the 95th percentile envelope (red line). Different colors represent different satellites.

To characterize the relationship between elevation and observable behavior and define the empirical fits, a large dataset of $\Delta_{t}C^{-}$ and $\Delta_{t}^{2}\phi$ values was compiled from both OS and FR scenarios, representing various conditions. Each index was visually inspected against elevation to identify general trends. Figure 4 illustrates the scatterplot of $\Delta_{t}C^{-}$ against the elevation. This graph presents data from a one-hour dataset collected on DOY 345 of 2024 in FR. The graph shows more dispersion at low elevations (left) and tighter clustering at higher elevations (right). A high density of points within ±1 m/s across all elevations is noted, representing nominal measurement conditions. This observation occurs because most epochs, even in forested environments, do not exhibit high peaks and experience only normal noise, causing the density to be consistently higher around the zero line. The 95th percentile envelope (red lines) quantifies the relationship between the index $\Delta_{t}C^{-}\;$ and the elevation, showing increased standard deviation of $\Delta_{t}C^{-}\;$ with lower elevations. Different colors on this plot indicate different satellites, and the plot is limited vertically to ±10 m/s, with crosses marking datapoints that are beyond this range.

A similar analysis was conducted for $\Delta_{t}^{2}\phi$ that revealed comparable elevation-dependent trends. However, to maintain conciseness, the corresponding figure is omitted. The consistent elevation dependence observed across both indices justifies using adaptive, elevation-scaled thresholds. These thresholds detect peaks by identifying when each detection index ($\Delta_{t}C^{-}$ and $\Delta_{t}^{2}\phi$) exceeds normal measurement behavior. Thresholds (τ) are computed per satellite and epoch using the general form, where x represents either detection indexes:

$$\tau_{x}=\;\bar{x}\pm K_{x}(\varepsilon )\cdot \sigma_{x}$$

where $\bar{x}$ and $\sigma_{x}$ denote the mean and standard deviation of the index, respectively over a 900-sample window, equivalent to 900 s or 15 min without data gaps; $K_{x}(\varepsilon )$ signifies a scaling term that modulates threshold tightness based on satellite elevation (ε) and is empirically computed separately for each index; the mean and standard deviation are computed after outlier removal (Section 3.4), with default values applied if samples are insufficient. Moreover, these samples are collected from past and current data only, with no future data used, allowing real-time implementation.

**Figure 5:**
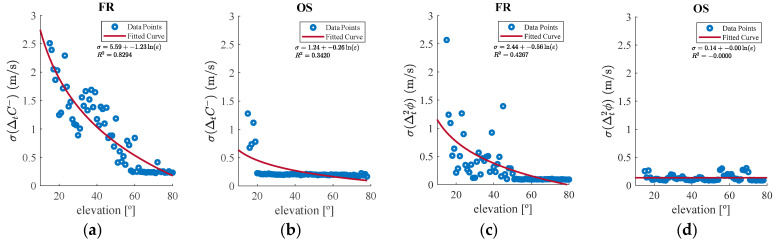
Standard deviation of (**a**) $\Delta_{t}C^{-}$ in FR, (**b**) $\Delta_{t}C^{-}$ in OS, (**c**) $\Delta_{t}^{2}\phi$ in FR, and (**d**) $\Delta_{t}^{2}\phi$ in OS as a function of satellite elevation.

To determine the $K_{x}(\varepsilon )$ terms for each index, all $\Delta_{t}C^{-}$ and $\Delta_{t}^{2}\phi$ measurements from FR and OS sites are grouped by integer elevation degrees, and standard deviations are computed for each group. Figure 5 depicts these statistics against elevation angles for both indices and environments. Unlike Figure 4, where all data plots were plotted, the data are grouped by integer elevation degrees to clarify trend visualization. The red line labeled “fitted curve” represents the optimal logarithmic fit equation for each case, with the corresponding equation and the coefficient of determination ($R^{2}$) displayed in the top-right corner of each plot.

These fitted curves are used to define the elevation-dependent threshold scale factors used in the algorithm. Since thresholds are environment-independent, a balanced approach is adopted. The resulting threshold expressions for $\Delta_{t}C^{-}$ and $\Delta_{t}^{2}\phi$ detection indices are defined as:

$$\tau_{C^{-}}=\bar{\Delta_{t}C^{-}\;}\pm \;K_{C^{-}}\cdot \sigma_{C^{-}}\;\;where\;K_{C^{-}}=1.1\;ln(\varepsilon )-0.2\;\;and\;\;K_{C^{-}}\in [0.5,0.7]$$

$$\tau_{\phi }=\bar{\Delta_{t}^{2}\phi }\pm K_{\phi }\cdot \sigma_{\phi }\;where\;K_{\phi }=0.6\ln\left(\varepsilon \right)+0.8\;and\;\;K_{\phi }\in [0.5,0.8]$$

These formulas capture the empirical trend while maintaining stability across the elevation range. Bounds are introduced to prevent thresholds from becoming overly restrictive at low elevations or excessively loose at high elevations.

### 3.4. Outlier Handling

Before applying the detection thresholds (Section 3.3), the $\Delta_{t}^{2}\phi$ and $\Delta_{t}C^{-}$ indices are adjusted to mitigate the impact of abrupt spikes or transient noise. Such outliers can inflate statistical metrics, such as the standard deviation, raising the detection thresholds and lowering sensitivity to true cycle slips.

To address this issue, the proposed algorithm includes a statistical outlier removal step applied before computing the local mean $\left(\bar{x}\right)$ and standard deviation ($\sigma_{x}$). Within each 900-sample window, the five largest-magnitude values are excluded from the statistical calculations. This value is selected empirically to balance robustness and responsiveness. By removing extreme values, regardless of actual cycle slips or strong noise peaks, the algorithm ensures that threshold computations remain representative of the signal behavior.

**Figure 6:**
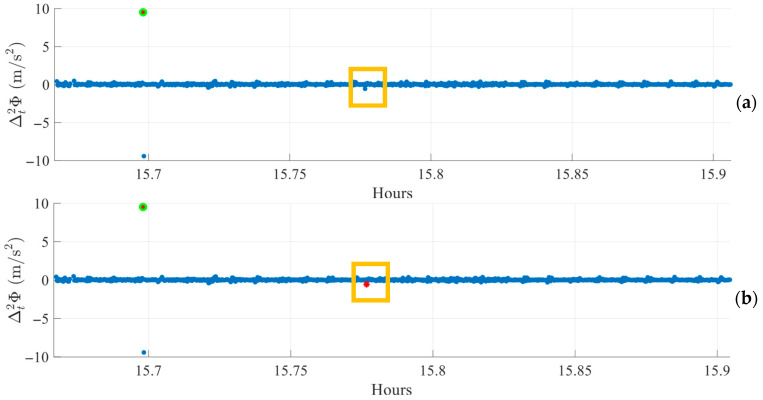
Example of peak detection in $\Delta_{t}^{2}\phi$ (**a**) before and (**b**) after outlier exclusion.

The effect of outlier filtering on peak detection is illustrated in Figure 6. Panel (a) shows the index $\Delta_{t}^{2}\phi$ before filtering, where a small peak around 15.8 h (highlighted in the yellow box) is visible but not detected because the threshold is biased upward by outliers, which increase the standard deviation. Panel (b) shows the same interval after outlier filtering. Here, the removal of large deviations lowers the threshold, enabling the apparent peak to be detected, which is marked with a red star. This step stabilizes the statistical measures used for thresholding and improves the algorithm’s ability to identify subtle cycle slips, even in environments subject to occasional large outliers.

## 4. Results and Analysis

This section evaluates the proposed cycle slip detection algorithm through experimental testing across multiple signal conditions. It evaluates case studies and statistical validation, providing both qualitative and quantitative performance data. All datasets are processed offline for reproducibility and detailed inspection while executing the algorithm using a configuration that fully preserves its real-time operational structure.

The evaluation comprises three components. Section 4.1 examines the temporal behavior of detection indices for selected satellite tracks under static conditions through case studies. Section 4.2 provides a statistical assessment using artificially injected cycle slips. Finally, Section 4.3 compares algorithm outputs against standard LLI flags from RINEX files to validate consistency with existing methods.

### 4.1. Visual Inspection in Static Environments

Static tests provide controlled conditions for evaluating the detection algorithm. This section examines representative cases from OS and FR environments to visually assess the dual-index detection logic and algorithm behavior under real-world signal conditions.

**Figure 7:**
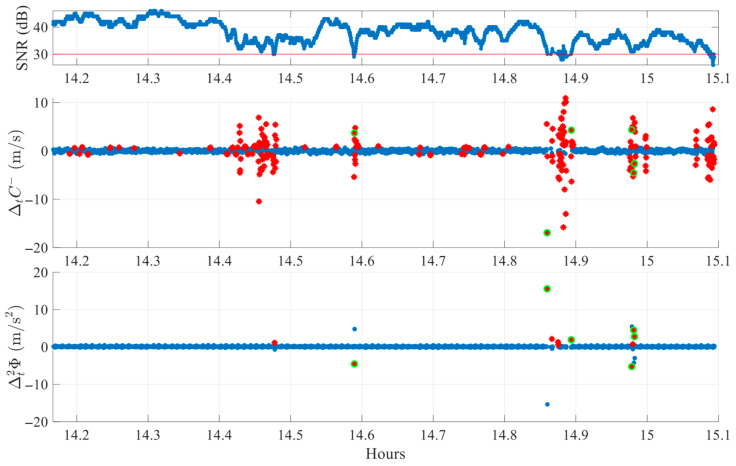
Example of cycle slip detection in FR environment (SV G29 on DOY 345, 2024). Red stars mark single-index crossings, while green circles denote simultaneous crossings classified as slips.

Each case is analyzed visually using a three-panel plot, as shown in Figure 7. The first subplot displays the SNR with a 30 dB reference line to indicate signal quality. The second and third subplots display the $\Delta_{t}C^{-}$ and $\Delta_{t}^{2}\phi$ detection indices, respectively. In these plots, red stars indicate epochs where a single index exceeds its adaptive threshold, while green circles represent epochs where both indices crossed their thresholds simultaneously. Only synchronized threshold crossings are flagged as potential cycle slip events.

Figure 7 illustrates an FR environment case, using data from satellite G29 on DOY 345 of 2024. The satellite is observed for one hour at an average elevation of 24.7°. Clear cycle slips are visible around 14.6 h, 14.9 h, and 15.0 h, where co-located peaks in both the $\Delta_{t}C^{-}$ and $\Delta_{t}^{2}\phi$ indices exceed their respective thresholds. Other peaks, such as those seen only in $\Delta_{t}C^{-}$ at 14.4–14.5 h or near 15.1 h, are not classified as slips since the second index remains within normal bounds. This finding confirms the algorithm’s selective behavior; detection requires agreement between both indices, in line with the theoretical definitions provided in Section 3.1. Similar rejections also occur for peaks present only in $\Delta_{t}^{2}\phi$, (e.g., near 14.5 h and 14.9 h), reinforcing the complementary nature of the dual-index logic.

**Figure 8:**
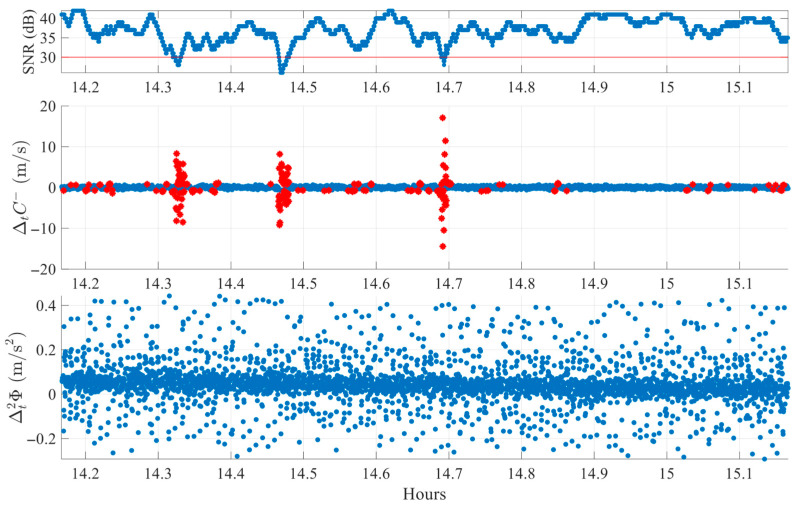
Example of cycle slip rejection in FR environment (SV E08 on DOY 345, 2024). Red stars mark single-index crossings, while green circles denote simultaneous crossings classified as slips.

Figure 8 presents another FR session case for satellite E08. Several pronounced deviations appear in $\Delta_{t}C^{-}\;$ near 14.3 h, 14.5 h, and 14.7 h, each exceeding the individual threshold. However, in the corresponding epochs, the $\Delta_{t}^{2}\phi$ index shows minimal variability with almost no peaks. The absence of synchronized peaks in both indices results in proper rejection of these epochs as non-slip events. Compared to Figure 6, this case more clearly demonstrates the total absence of peaks in $\Delta_{t}^{2}\phi$ index. These findings highlight a critical advantage of the proposed dual-index approach in effectively discriminating between possible genuine cycle slips and measurement artifacts commonly induced by noise fluctuations in obstructed environments.

**Figure 9:**
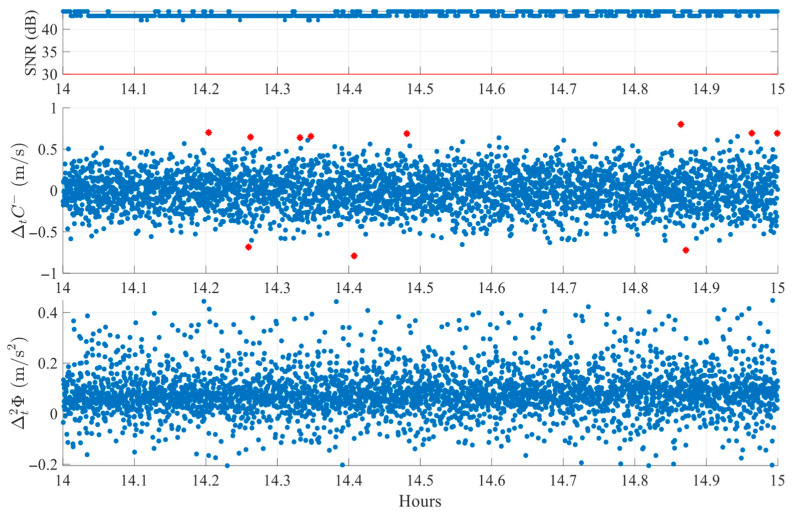
Example of true negative case in OS environment (SV E07 on DOY 345, 2024). Red stars mark single-index crossings, while green circles denote simultaneous crossings classified as slips.

Figure 9 illustrates the algorithm performance under ideal signal conditions using data from satellite E07 observations at a high elevation angle (~65°) in the OS environment. The SNR values remained above 40 dB throughout the measurement period, which indicates that the reception conditions were optimal. Both detection indices remain stable throughout the observation period, with minimal deviation and no peaks exceeding thresholds. The algorithm correctly avoids any detections, indicating stable operation and a low risk of false positives under favorable signal conditions. This case with no peaks supports the validity of the threshold calibration, ensuring that nominal variations are not mistakenly flagged as slips.

Across all static cases, the dual-index detection logic consistently differentiates between potential slips and isolated anomalies. The FR environment exhibits greater measurement variability, particularly in $\Delta_{t}C^{-}\;$ due to higher susceptibility to code noise. However, the adaptive thresholds accommodate this variability, preserving the algorithm’s sensitivity to actual events while minimizing misclassifications.

### 4.2. Statistical Evaluation with Artificial Slips

To quantitatively assess the algorithm’s accuracy and robustness beyond visual inspection, artificially injected cycle slips are introduced into real carrier-phrase datasets from static GNSS sessions. This controlled approach operates under known ground truth conditions. Ten one-hour satellite datasets, previously visually confirmed to contain no natural cycle slips, are selected from the static OS measurement campaigns. The use of real, unfiltered data ensures realistic signal characteristics, although the presence of measurement white noise limits the minimum detectable slip size to five cycles. Slips smaller than this threshold cannot be reliably distinguished from measurement noise and are therefore excluded from the tests.

Artificial slips are injected by randomly selecting epochs and amplitudes within defined constraints. At each selected epoch, integer carrier cycles are added or subtracted from the carrier-phase time series. Slip injections begin from the third epoch, as double differencing requires at least two previous epochs for calculation. Two parameters are varied in the tests: cycle slip magnitude and occurrence frequency. The cycle slip magnitude is categorized into 5–10 (subtle slips), 10–15 (moderate slips), 15–20 (pronounced slips), and 20–50 cycles (large slips). The occurrence frequency is set at four injection rates: 5, 15, 30, and 60 events per hour per satellite, representing scenarios from sparse to dense slip occurrence.

For statistical reliability, the procedure was repeated ten times for each parameter combination, across ten satellites, four slip magnitudes, and four slip rates. This resulted in 100 tests per slip magnitude–rate combination (10 satellites × 4 magnitudes × 4 rates × 10 repetitions), yielding a total of 1600 artificial slip tests. These large-scale trials provide robust statistics for evaluating detection accuracy. The detection success rates obtained for each parameter combination are summarized in Table 2. A detection is considered successful only if the algorithm reports a slip at the exact epoch where it is injected.

No false positives are observed in any configuration. Consistently high detection rates (exceeding 96%) are observed across all tested configurations, with slight degradation in performance under the most challenging conditions, such as smaller slips at higher injection rates. Perfect detection is achieved for almost all slip magnitudes under lower-frequency injections, confirming optimal performance when temporal separation allows complete index recovery between events.

Analysis of the results reveals that detection performance depends on slip magnitude and injection rate. In terms of slip magnitude, detection performance remains exceptionally high across all ranges, but smaller slips are more affected by dense injections. For example, in the 5–10 cycle range, the detection probability decreases from 99.60% at five events per hour to 96.68% at 60 events per hour, representing a relative drop of 2.92%. In contrast, slips in the 20–50 cycles experience a much smaller reduction, from 100% to 99.07%, corresponding to a 0.93% decrease. This finding confirms that smaller slips have higher susceptibility to degradation under dense injection conditions, as they are often masked by measurement noise.

In Table 2, larger slips consistently achieve higher detection probabilities than smaller ones, though the differences are minimal when events are infrequent. For instance, at five events per hour, the smallest slips are detected at 99.60% and the largest at 100%, a gap of only 0.40%. However, at 60 events per hour, detection rises from 96.68% for the smallest slips to 99.07% for the largest, a difference of 2.39%. These results demonstrate that slip magnitude has a greater impact on performance when events occur in rapid succession, whereas under sparse conditions, detection rates are nearly independent of magnitude.

Overall, these patterns indicate that both magnitude and occurrence rate affect detection, but their influence is interdependent. Larger slips are reliably detected under all tested conditions, whereas smaller slips are more vulnerable to noise and high event density. The statistical results confirm the algorithm’s high accuracy in detecting cycle slip events while maintaining low false alarm positives. Although performance degrades with smaller slip magnitudes and higher injection rates, it remains suitable for practical applications.

**Figure 10:**
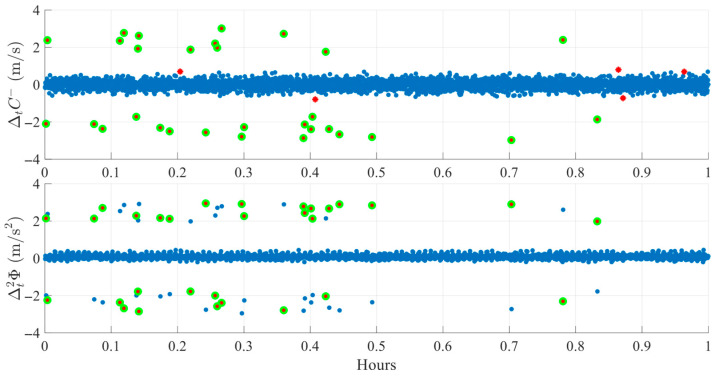
Example of an artificial cycle slip detection test.

Figure 10 presents the testing procedure of one representative case with 30 injection slips per hour of 10–15 cycles. The figure does not represent overall performance trends but illustrates how artificially injected slips appear in the carrier-phase data and how the algorithm detects them. The figure contains two subplots, one for each index. Red stars mark peaks, while green circles indicate detected cycle slips. In this example, all 30 cycle slips are correctly detected, with visual inspection confirming 30 cycle slips, aligning in time with the artificial cycle slip injections.

### 4.3. LLI Correlation Study

To further assess the proposed algorithm, it was benchmarked against conventional slip indicators by comparing its detections with LLI values recorded in the original RINEX observation files. While LLI flags do not constitute absolute ground truth, particularly in low-cost receivers where tracking loop sensitivities vary, they serve as a valuable reference for identifying epochs where the receiver flagged potential cycle slips or tracking discontinuities.

In RINEX files, LLI = 1 indicates a possible cycle slip, LLI = 2 denotes a half-cycle ambiguity, and LLI = 3 signifies both conditions. Since flagged LLI epochs often occur in consecutive sequences, the comparison was performed using a region-based approach rather than on individual epochs. Contiguous non-zero LLI intervals were grouped into “LLI boxes” to represent sustained periods of signal instability rather than single moments.

**Figure 11:**
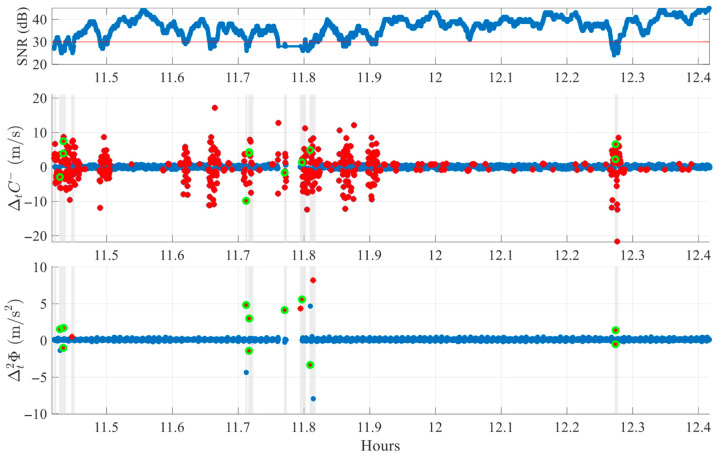
Comparison between algorithmic cycle slip detections and RINEX LLI flags for SV G30 on DOY 070 of 2025 in FR environment. Red stars mark single-index crossings. Green markers indicate detected slips. Shaded regions denote LLI boxes where LLI ≠ 0.

Figure 11 illustrates this approach using data from SV G30 in the FR environment, with detected slips (green markers) and LLI boxes (gray rectangles). Most detections are located within these intervals, some aligning exactly with LLI = 1 epochs, while others fall slightly outside box boundaries, reflecting subtle time differences. Some LLI-marked epochs have no cycle slip detections, either due to signals too weak to be considered a peak or events appearing in only one index.

**Figure 12:**
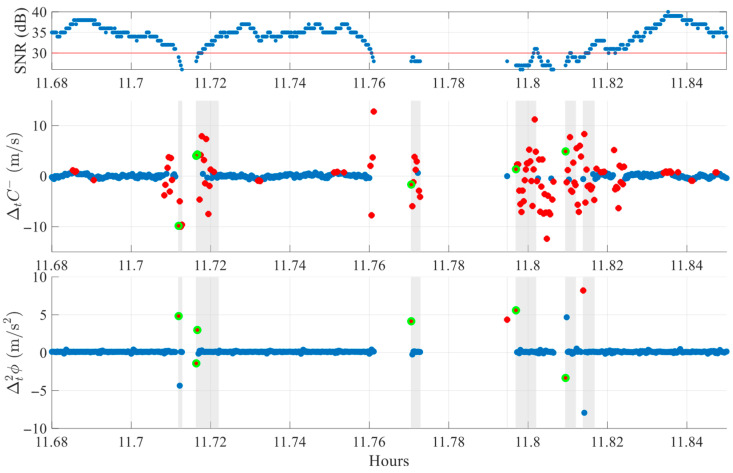
Comparison between algorithmic cycle slip detections and RINEX LLI flags for SV G30 on DOY 070 of 2025 in FR environment zoomed at 11.7–11.85 h. Red stars mark single-index crossings. Green markers indicate detected slips. Shaded regions denote LLI boxes where LLI ≠ 0.

To better visualize LLI box characteristics, Figure 12 zooms into the 11.7–11.85 h interval. Boxes of different durations appear depending on the number of consecutive flagged epochs. In most cases, at least one cycle slip detection is present within each box.

Analysis of all the collected data obtains four performance indicators. About 58.16% of all LLI boxes contain at least one detected cycle slip, showing that the method responds to many receiver-flagged instability periods. In addition, 87.93% of all detected cycle slips occur within LLI boxes, indicating strong spatial agreement despite algorithmic independence from receiver tracking logic. At the epoch level, 64.04% of detected slips coincided exactly with LLI = 1 or 3 flags, while 37.20% of LLI = 1 or 3 epochs triggered algorithmic cycle slip detections.

These results validate that the proposed method identifies many of the same discontinuities as flagged by the receiver. With nearly 88% of detected slips occurring within LLI boxes and over 64% of detections matching LLI = 1 or 3 epochs exactly, the algorithm generally agrees with the receiver’s assessment of signal instability and with the most relevant receiver slip flags, respectively.

Furthermore, the algorithm is more selective than LLI, flagging 37% of LLI = 1 or 3 epochs. This selectivity should be regarded as a strength: the algorithm avoids reacting to weak or ambiguous events that often cause false alarms. In practice, this ensures that only slips with clear signatures are detected, providing higher reliability compared with raw LLI flags. Similarly, 42% of LLI boxes contain no detections, suggesting that not all receiver-flagged instability periods correspond to observable cycle slips. This selective filtering ensures that only robustly identified slips with strong signatures in both observables are detected, while weak, noisy, or ambiguous events are ignored.

## 5. Discussion and Conclusions

This study proposes a two-step cycle slip detection algorithm for single-frequency GNSS receivers, combining $\Delta_{t}C^{-}$ and $\Delta_{t}^{2}\phi$ indices to identify slips with high confidence. The method operates in real time using only past and current data and requires no user position solutions, dual-frequency data, or external sensor aids, making it suitable for low-cost and embedded systems. Robustness is enhanced through adaptive, elevation-dependent thresholds and outlier filtering, while mirrored-peak suppression in $\Delta_{t}^{2}\phi$ indices reduces false positives caused by second-order differencing.

Experimental tests on both static and artificial scenarios confirm consistent detection performance. In OS datasets, true slips are detected reliably, while in FR datasets, natural signal variations do not trigger false alarms. Artificial slip tests demonstrated robustness, achieving over 98% detection for slips larger than 10 cycles even under dense injection (60 events per satellite per hour), and 100% detection in moderate-to-high magnitude, low-rate cases, all without false positives. It should be noted, however, that the present validation was conducted under nominal ionospheric conditions, and slips smaller than 5 cycles remained difficult to separate from measurement noise in unfiltered single-frequency data. The algorithm detects a large proportion of receiver-flagged instability periods, yet remains more selective than standard LLIs: nearly 88% of detections fall within LLI intervals, and over 64% correspond exactly to epochs with LLI = 1 or 3. This selective behavior ensures stable performance even in noisy environments.

Given its lightweight and real-time nature, the framework is well-suited for integration into low-cost GNSS receivers, IoT devices, and embedded navigation platforms, where computational efficiency and robustness are essential. This makes the method immediately useful for a wide range of positioning applications.

Overall, the results demonstrate that the proposed method offers a reliable, lightweight, and real-time-capable solution for single-frequency cycle slip detection, with strong potential for integration into practical GNSS applications.

## Figures and Tables

**Table 1:** Classification logic for two-step cycle slip detection based on cross-validation of $\Delta_{t}C^{-}$ and $\Delta_{t}^{2}\phi$ indices.

$\Delta_{t}C^{-}$ Peak	$\Delta_{t}^{2}\phi$ Peak	Classification
Yes	Yes	Confirmed Cycle Slip
Yes	No	Rejected (Not CS)
No	-	No Detection

**Table 2:** Probability of detection of artificial cycle slips under varying size and occurrence rate conditions.

		Cycle Slip Occurrence [Cycles/Hour]			
		5	15	30	60
**Cycle Slip** **Magnitude** **[cycles]**	**5~10**	99.60	99.00	98.47	96.68
	**10~15**	100	99.67	98.97	98.27
	**15~20**	100	99.87	99.30	98.28
	**20~50**	100	99.87	99.77	99.07

## Data Availability

The raw data supporting the conclusions of this study will be made available by the corresponding author on request.
